# Abnormal vestibular-evoked myogenic potentials as a risk factor for unpredicted falls in spinocerebellar ataxia: a preliminary study

**DOI:** 10.1007/s00415-024-12195-6

**Published:** 2024-01-27

**Authors:** Seo-Young Choi, Kwang-Dong Choi, Jae-Hwan Choi, Ji-Soo Kim

**Affiliations:** 1 Department of Neurology, Pusan National University Hospital, Pusan National University School of Medicine and Biomedical Research Institute, Busan, South Korea; 2 Department of Neurology, Research Institute for Convergence of Biomedical Science and Technology, Pusan National University Yangsan Hospital, Pusan National University School of Medicine, Yangsan, South Korea; 3 Department of Neurology, Dizziness Center, and Clinical Neuroscience Center, Seoul National University Bundang Hospital, 173-82 Gumi-ro, Bundang-gu , Seongnam-si, Gyeonggi-do 13620 South Korea; 4 Department of Neurology, Seoul National University College of Medicine, Seoul, South Korea

**Keywords:** Spinocerebellar ataxia, Fall, Vestibular function test, Otolith function test, Vestibular evoked myogenic potential

## Abstract

**Objective:**

This study aimed to correlate the symptoms and signs with the findings of laboratory vestibular function tests in patients with spinocerebellar ataxia (SCA).

**Method:**

We retrospectively recruited 26 patients with SCA (9 men, median age: 52, age range: 21–67). Assessments included Dizziness Handicap Inventory, EuroQoL Five-Dimension, symptom questionnaires manifesting during walking in daily life, the Scale for the Assessment and Rating of Ataxia (SARA), and vestibular function tests including 3D video-oculography, video head impulse test, subjective visual vertical, and cervical and ocular vestibular evoked myogenic potentials (VEMP).

**Results:**

Cross-analyses revealed that the patients with VEMP abnormalities showed higher SARA (*p* = 0.014) and prevalence of unpredictable falls (*p* = 0.046). The patients with SCA1 more frequently had unpredictable falls (75%, *p* = 0.038) and VEMP abnormalities (88%, *p* = 0.001) compared to SCA2 (29% falls, 17% VEMP abnormalities) and SCA6 (no falls or VEMP abnormalities).

**Conclusion:**

Abnormal VEMPs are strongly associated with unpredicted falls in patients with SCA, particularly in those with SCA1. Impaired processing of otolithic information may contribute to falls in SCAs, and VEMP may help identifying the patients with a risk for unpredicted falls and preventing fall-related injuries in SCA. Limited number of patients with lower SARA scores warrant further confirmatory studies.

## Introduction

The autosomal dominant spinocerebellar ataxias (SCA) are neurogenerative disorders characterized by progressive cerebellar ataxia due to Purkinje cell degeneration. [1] The most common forms, SCA type 1, 2, 3, and 6, are caused by expansions of CAG repeats encoding the polyglutamine tracts within the several unrelated genes, ataxin1, ataxin2, ataxin3, and the gene encoding the alpha1 A subunit of voltage-gated calcium channel. [1–3] Even though SCAs are genetically and clinically heterogenous, cerebellar and brainstem ocular motor signs are common to SCAs. [4, 5] Evaluation of eye movements such as nystagmus, saccades, smooth pursuits, and the vestibulo-ocular reflex (VOR), each characterized by unique neural pathways, [6] may aid in specification of each SCA subtype. [7] Furthermore, since eye movements can be recorded and quantified, abnormal eye movements may serve as a marker for disease progression and therapeutic efficacy and guide genetic testing. [7]

Previous studies have mostly concentrated on associations among the CAG repeat numbers, ataxia scores, and neuroimaging abnormalities in SCA. [2, 8] However, the ataxia score alone cannot fully represent the quality of life (QoL), especially unpredicted falls that significantly affect the QoL. [9–12]

Despite the efforts to find out medication targeting the pathophysiological mechanisms of SCAs, a definitive cure remains elusive in these disorders. [13, 14] In this context, researches focusing on improving the QoL throughout a patient's life are essential. [15] Thus, it would be valuable to identify objective neurological signs that correlate with the subjective symptoms or QoL, potentially serving as the clinical markers for guidance of therapeutic intervention. The objective of this study is to illuminate any correlation between the objective findings of ocular motor and vestibular function tests and the symptoms and QoL in patients with SCAs.

## Methods

### Patients

Two authors retrospectively reviewed the medical records of 37 patients with genetically confirmed autosomal dominant SCA at the dizziness clinic of Pusan National University Hospital from January 2019 to March 2022. We finally recruited 26 (9 men, median age: 52, age range: 21–67) for analyses after excluding 11 patients with incomplete evaluation due to a bed-ridden state (n = 4), a follow-up loss (n = 4), refusal of the testing (n = 2), and a previous history of vestibular neuritis (n = 1). The subtypes of SCA included SCA1 in eight from four families, SCA2 in 14 from eight families, and SCA6 in four from three families. There were no differences in age, sex, and disease duration among the subtypes (Table 2).

### Standard protocol approvals, registration, and patient contents

This study followed the tenets of the Declaration of Helsinki and was performed according to the guidelines of the Institutional Review Board of Pusan National University Hospital (2108–001-105).

### Evaluation of ataxia and scoring for symptoms and quality of life

The assessments for cerebellar ataxia were based on the Scale for the Rating and Assessment of Ataxia (SARA) that was scored by the sum of the gait, stance, sitting, speech disturbance, finger-chase, nose-finger test, fast alternating hand and heel-shin movements. [5] Self-administered questionnaires for evaluation of dizziness and health-related QoL included the dizziness handicap inventory (DHI) [16, 17] and the five-level EuroQoL five-Dimension (EQ-5D-5L). [18] To assess the symptoms during walking in daily life, the following yes or no questions were also added; "Q1: Have you had unpredictable falls?" "Q2: Do you feel more dizzy in moving conditions, such as in a moving elevator or escalator?" "Q3: Do you feel a pulling sensation to one side while walking (lateropulsion)?".

### Oculography and video head impulse tests

Eye movements were recorded at a sampling rate of 60 Hz using 3D-video-oculography (VOG, SLMED, Seoul, Korea). Spontaneous nystagmus (SN) with or without visual fixation, gaze-evoked nystagmus (GEN), and horizontal saccades and smooth pursuit were pursued following the methods described previously in detail. [19] Head and eye movements were also recorded during head impulses using a video-based equipment (vHIT, SLMED, Seoul, South Korea). The detailed methods and normative data on gain of the vestibulo-ocular reflex (VOR) for six semicircular canals (SCCs) were described previously. [20]

### Cervical and ocular vestibular evoked myogenic potentials, and subjective visual vertical

The detailed methods for recording cervical and ocular vestibular evoked myogenic potentials (VEMPs) and the subjective visual vertical (SVV) were described previously. [20, 21] VEMPs were recorded using a Nicolet Viking Select unit (Nicolet Biomedical, Madison, WI, USA) on the same day. Cervical VEMP (cVEMP) was recorded from the ipsilateral sternocleidomastoid muscle (SCM) that was activated by elevating and turning the head contralaterally in the supine position. Electromyographic activities of the SCM were simultaneously recorded using a surface electrode from the upper half of the SCM with a ground electrode on the forehead and a reference electrode over the upper sternum. A short alternating tone burst (110 dB nHL; 121.5 dB SPL; 500 Hz; ramp = 2 ms; plateau = 3 ms) was given at 2.1 Hz monoaurally using a headphone. During the recording, the EMG activities of the SCM were also monitored and digitized at 1 kHz using an analog-to-digital converter (NI PCI-4461, National Instruments, Austin, TX, USA). The LabVIEW program (National Instruments) was used to analyse the peak-to-peak amplitudes and calculate the mean tonic activation during the recording. The absolute cVEMP amplitude was then normalized against the mean tonic activation of the SCM during the recording. [21] We confirmed that none of the patients had the conditions leading to conductive hearing loss, such as middle ear infections, and no patients reported subjective hearing impairments. To record ocular VEMP (oVEMP), patients sat with their head upright while fixating on a target that was displaced on the wall, more than 2 m away from the eyes, with an angle of gaze more than 20° upward. Electromyographic activities were recorded using a surface electrode placed on the infra-orbital ridge 1 cm below the center of the lower eyelid. The reference electrode was attached 2 cm below the active electrode, and the ground electrode on the forehead. Bilateral oVEMPs were induced tapping the hairline at AFz up to 60 times using an electric reflex hammer (VIASYS Healthcare, CA, USA). The latency of cVEMP was measured as the time when the positive wave (p13) at around 13 ms and the negative wave (n23) at around 23 ms appeared, and the amplitude between these two waves was normalized to the SCM contraction. For oVEMP, the latency of the negative wave at around 10 ms (n10) and the amplitude between n10 and the subsequent positive wave were measured. We compared the VEMPs of the patient group with those of 26 age-matched control (Median age = 53, IQR 40–58, *p* = 0.096). Abnormal VEMPs were defined as no responses to stimulation or asymmetry of the amplitudes > 20%. [20, 21]

The SVV tilt was measured while seating the patient in a darkroom. The patients were instructed to align a randomly tilted rod (80 cm long and 0.3 cm wide) vertically using a joystick at least five times per each session. The normal value of SVV is ± 3° during binocular viewing (a negative value indicates a counterclockwise rotation). [20, 21]

### Statistical analysis

Statistical analyses were performed using SPSS (version 23.0; SPSS, Chicago, IL). Quantitative values are presented as the median (IQR, first—third quartile) or mean ± standard deviation (SD), and the qualitative values as the frequency (%, percent). The Kruskal–Wallis or Mann–Whitney test was used for the continuous variables and Chi-square or Fisher’s exact tests for the nominal ones. When comparing the continuous variables among the groups, we employed either the student t or Mann–Whitney test. The significance level was set at *p* < 0.05.

## Results

Thirteen (50%) Patients reported moderate dizziness with the median DHI score at 32 (IQR, 11–63) and the median EQ-5D-5L score at 10 (IQL, 8–14) (Table 1). Almost all the patients (25/26, 96%) showed gait abnormalities with the score more than 2 on the gait subdomain of SARA, which indicates that the patients could not complete more than 10 tandem steps. The SARA, DHI, and QoL did not differ among the subtypes of SCA.

**Table 1:** Summary of clinical profiles and vestibular findings

Clinical profiles and Ataxia scale		
Age, median (range), year		52 (21—67)
Men, n (%)		9 (35)
Symptom onset to evaluation, median (IQR), year		6 (3—10)
Type, n (%)	SCA 1	8 (31)
	SCA 2	14 (54)
	SCA 6	4 (15)
SARA, median (IQR)		10 (5—11)
Subjective symptoms		
DHI, median (IQR)		32 (11—63)
EQ-5D-5L, median (IQR)		10 (8—14)
Unpredicted falling, n (%)		10 (38)
Dizziness in moving conditions, n (%)		2 (8)
Lateropulsion while walking, n (%)		10 (38)
Ocular motor and vestibular findings		
Hypermetric saccades, n (%)		12 (46)
Saccadic slowing, n (%)		15 (58)
Gaze-evoked nystagmus, n (%)		11 (42)
Impaired smooth pursuit, n (%)		26 (100)
Abnormal VEMPs, n (%)		9 (35)
	Abnormal cervical VEMPs, n (%)	7 (27)
	Abnormal ocular VEMPs, n (%)	6 (23)
Abnormal vHIT gain	Horizontal canal, n (%)	10 (38)
	Vertical canal, n (%)	7 (27)
SVV, Median (IQR)		0 (− 1—1)

*DHI* dizziness handicap inventory, *EQ-5D-5L* EuroQoL Five-Dimension, *SARA* scale for the assessment and rating of ataxia, *SCA* spinocerebellar ataxia, *SVV* subjective visual vertical, *VEMP* vestibular evoked myogenic potential, *vHIT* video head impulse test, *VOR* vestibulo-ocular reflex

About one third of patients (10/26, 38%) also reported unpredicted falls or gait unsteadiness. Of interest, patients with SCA1 showed a higher prevalence of unpredicted falls (6/8, 75%, *p* = 0.038, Table 2). In contrast, only two patients (8%) reported dizziness in a moving elevator or escalator (Table 1).

**Table 2:** Comparison of findings among the patients with SCA1,2, and 6

		SCA1 (n = 8)	SCA2 (n = 14)	SCA6 (n = 4)	*p* value
Age, median (IQR), year		54 (49–56)	48 (38–58)	45 (34–56)	0.675
Men, n (%)		3 (38)	4 (29)	2 (50)	0.372
Symptom onset to evaluation, median (IQR), year		6 (3 -10)	7 (4–9)	7 (3–10)	0.840
DHI, median (IQR)		47 (21–68)	17 (10–48)	49 (36–62)	0.526
EQ-5D-5L, median (IQR)		14 (8–16)	9 (7–12)	12 (10–14)	0.473
Other symptoms, n (%)	Unpredicted falling	6 (75)	4 (29)	0 (0)	0.038
	Dizziness in moving conditions	1 (13)	0 (0)	1 (25)	0.203
	Lateropulsion while walking	1 (13)	6 (43)	3 (75)	0.098
SARA, median (IQR)		11 (8–20)	11 (7–11)	4 (2–6)	0.091
Ocular motor or vestibular dysfunction					
Hypermetric saccades, n (%)		5 (63)	4 (29)	3 (75)	0.222
Saccadic slowing, n (%)		2 (25)	13 (93)	0 (0)	< 0.0001
Gaze-evoked nystagmus, n (%)		2 (25)	5 (36)	4 (100)	0.046
Impaired smooth pursuit, n (%)		8 (100)	14 (100)	4 (100)	1.000
Decreased gain during vHIT, n (%)	Horizontal canals	5 (63)	2 (14)	3 (75)	0.022
	Vertical canals	2 (25)	3 (21)	2 (50)	0.519
Abnormal VEMPs, n (%)		7 (88)	2 (17)	0 (0)	0.001
	Cervical VEMP	6 (75)	1 (7)	0 (0)	0.001
	Ocular VEMP	5 (63)	1 (7)	0 (0)	0.004
Abnormal SVV, median (IQR)		0.1 (− 0.6–0.3)	− 1.0 (− 2.5–0.4)	0.5 (0.1–0.9)	0.253

*DHI* dizziness handicap inventory, *EQ-5D-5L* EuroQoL Five-Dimension, *SARA* Scale for the Assessment and Rating of Ataxia, *SCA* spinocerebellar ataxia, *SVV* subjective visual vertical, *VEMP* vestibular evoked myogenic potential, *vHIT* video head impulse test, *VOR* vestibulo-socular reflex

### Ocular motor and vestibular function tests

Detailed description of the ocular motor findings is provided in Table 1. No patients exhibited SN with or without fixation. However, all patients showed cerebellar ocular motor dysfunction that included GEN, impaired smooth pursuit, or hypermetric saccades. GEN was observed in all patients with SCA6 (*p* = 0.040). Slowing of horizontal saccades was observed in 15 (58%) patients and was more prevalent in patients with SCA2 (13/14, 93%, *p* < 0.0001, Table 2).

The head impulse gain of the VOR was decreased for the horizontal semicircular canal (HC, 0.89 ± 0.10 vs. 0.94 ± 0.05, *p* = 0.004, *t*-test), but was normal for the vertical semicircular canals (0.95 ± 0.11 vs. 0.96 ± 0.04, *p* = 0.427, *t*-test). Overall, 14 patients (54%) showed a subnormal gain for at least one of the semicircular canals, 38% (10/26) for the HC and 27% (7/26) for the vertical canals. The reduced gain for the HC was less commonly observed in patients with SCA2 (2/12, *p* = 0.022).

Nine patients (35%) exhibited abnormal oVEMP (n = 6, 23%) or cVEMP (n = 7, 27%) (Table 1). Of them, bilateral absence of responses was observed in seven, oVEMP in five and cVEMP in five. Two other patients with abnormal cVEMP were marked by a unilateral absence of wave formation, one (SCA1) of them with an asymmetry oVEMP at 36%.

Most patients with SCA1 (7/8, 88%) demonstrated various patterns of abnormal VEMPs [abnormal cVEMP in 6 (75%, median age 54, range 43–65), abnormal oVEMP in 5 (63%, median age 56, range 43–65)] while only two patients (14%) with SCA2 and none of the patients with SCA6 showed abnormal VEMP (Table 2).

### Correlation between the symptoms and ocular motor/vestibular dysfunction

**Fig. 1:**
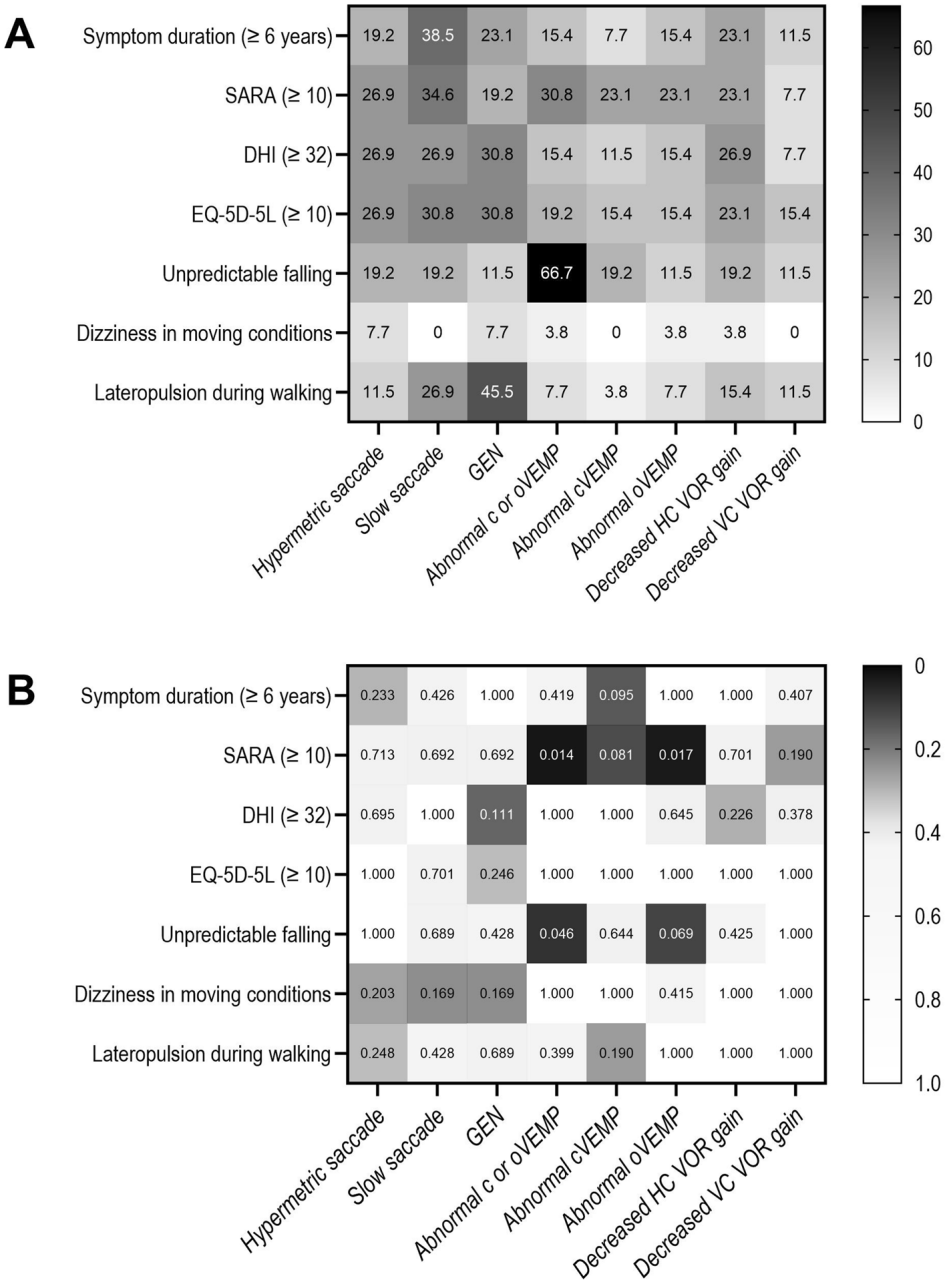
The frequency (**A**) and statistical significance (**B**) of ocular motor/vestibular dysfunction in patients with increased (median or higher) scores during each survey. The numbers in the small squares represent the proportion (**A**) and *p*-value obtained with Fisher’s exact test for cross-analysis (**B**). Darker shades in each square indicate higher proportion and lower *p*-values. *DHI* dizziness handicap inventory, *EQ-5D-5L* EuroQoL Five-Dimension, *GEN* gaze evoked nystagmus, *HC* horizontal semicircular canal, *SARA* Scale for the Assessment and Rating of Ataxia, *SCA* spinocerebellar ataxia, *SVV* subjective visual vertical, *VC* vertical semicircular canal, *VEMP* vestibular evoked myogenic potential, *vHIT* video head impulse test, *VOR* vestibulo-ocular reflex)

In the cross-analyses of the variables (Fisher's Exact Test), the patients with abnormal cVEMP or oVEMP showed a higher SARA score (*p* = 0.014) and a history of unpredicted falls (*p* = 0.046). Especially, the patients with abnormal oVEMP showed elevated SARA scores (*p* = 0.017) (Fig. 1B).

## Discussion

**Fig. 2:**
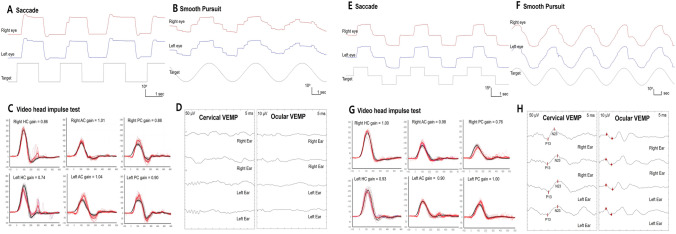
Representative ocular motor and vestibular dysfunction in each patient with spinocerebellar ataxia type 1 and 2 (**A**–**D**). A 54-year-old woman with spinocerebellar ataxia (SCA) type 1 shows hypermetric horizontal saccades (**A**) and impaired smooth pursuit (**B**). Video head impulse tests (vHIT) show decreased gain of the vestibulo-ocular reflex (VOR) for left horizontal semicircular canal (**C**). Cervical and ocular vestibular evoked myogenic potentials (VEMPs) show no responses bilaterally (**D**). **E**–**H** A 40-year-old woman with SCA2 shows slow horizontal saccades (**E**) and impaired smooth pursuit (**F**). In contrast, the findings of vHIT (**G**), and ocular and cervical VEMPs are normal (**H**)

We found a significant correlation between the VEMP abnormalities and unpredicted falls in patients with SCA, especially SCA1 Fig. 2.

Previous studies have found falls as a critical detrimental factor for the QoL in patients with SCA. [10, 11] A retrospective analysis reported falls in nearly 70% of 228 patients with SCA, having resulted in significant trauma in approximately 70% of them. [11] The factors associated with a higher likelihood of falls included elevated SARA scores, longer disease duration, presence of pyramidal and extrapyramidal signs, and SCA3. [11] The authors suggested that the leg dystonia, which is more prevalent in SCA3, could have contributed to falls. [11] In our study, the prevalence of falls was rather low (38%) compared to that of the previous study. This may be attributed to the unpredicted nature of falls defined in our study. Otherwise, exclusion of the patients in more advanced stage may explain the low prevalence.

While falls may occur due to diverse causes, otolithic dysfunction is an independent predictor. [22] VEMPs can evaluate central as well as peripheral otolithic function. [21–24] Even though the relationship between falls and VEMP abnormalities in SCA requires further elucidation, it has been the subject of studies in patients with parkinsonism. [14, 25, 26] Vestibular dysfunction, especially delayed responses or reduced amplitude of oVEMP or cVEMP, is associated with frequent falling in patients with idiopathic Parkinson's disease and atypical parkinsonism. [14, 25] Since central otolithic projections ascend via the medial longitudinal fasciculus and descend via the medial vestibulospinal tract in the brainstem, abnormal VEMPs indicate altered processing of the otolithic information in the brainstem due to neurodegeneration in these disorders. [25] Thus, in conjunction with bradykinesia and rigidity, impaired otolithic information may lead to frequent falls in patients with Parkinson's disease. [25] Furthermore, the patients with progressive supranuclear palsy also show a decreased gain of the linear VOR and reduced amplitude of cVEMP, which may contribute to the postural instability and falls frequently observed in this disorder. [26]

A few studies on otolithic dysfunction have shown controversial results in SCA3. [27, 28] A study of 18 patients with SCA3 did not show any difference in the latency and amplitude of cVEMP between the patients and controls, but found a decrease in the VOR gain for six SCCs during vHIT. [27] On the contrary, another study found abnormalities of either oVEMP or cVEMP in 93% (13/14) of patients with SCA3, [28] which may be explained by involvement of the vestibular nuclei in SCA3. [27] These differences could be attributed to individual variation or dissimilar disease severity of the patients participated in those studies. [27]

In our study, we observed characteristic ocular motor findings for each subtype of SCAs as previously reported, such as slow saccades for SCA2 [7] and GEN with impaired VOR gain for SCA6. [29] Prior studies have shown that the ocular motor findings are more variable in SCA1. [4, 30] Our study could not identify distinctive ocular motor findings specific for SCA1, either.

However, we found significantly higher prevalence of unpredicted falls and VEMP abnormalities in SCA1 than in other subtypes. Previous pathologic studies also documented distinct involvements of the vestibular nuclei, vestibulocerebellum, and dentate nuclei in SCA1 while those were spared in SCA2. [31] The lateral and superior vestibular nuclei are more severely affected than the medial vestibular nuclei in the advanced stages. [32] These findings may explain the frequent falls and VEMP abnormalities observed in SCA1 compared to SCA2 even though the disease duration was similar between the groups. Likewise, the VOR gains were also subnormal during the rotatory chair and caloric tests [4] and vHIT in SCA1. [33] In contrast, the VOR and VEMPs are mostly preserved in SCA2 while saccadic slowing was most prominent. [7, 33, 34] These findings also indicate a relative sparing of the vestibular structures and a predilection of the premotor structures for horizontal saccades, which was also confirmed in pathologic studies in SCA2. [31, 32] The preserved VEMPs in SCA6 are consistent with pure cerebellar involvement and sparing of the brainstem in this disorder while GEN and impaired high-frequency VOR are mostly due to neurodegeneration involving the flocculus. [29, 35]

Our study has several limitations. First, due to the relatively small number of patients included, particularly in SCA6, the differences in scores observed across the subtypes may have been underestimated. Moreover, we enrolled the patients with relatively lower SARA scores, who were capable of completing all the vestibular function tests. This implies that individuals with higher SARA scores, not represented in this study, may have a greater propensity for falls. The lack of SCA3 patients in our study is also a limitation. This absence precludes a comparison with the previous studies on the prevalence of falls in SCAs, underscoring the need for future researches that involve SCA3 patients with a large scale cohort or focuses on each subtype of SCAs for more comprehensive analyses.

## Conclusion

The unpredicted falls in SCA may be related to impaired processing of otolithic information, especially in type 1. The higher prevalence of unpredicted falls and abnormal VEMPs in SCA1 suggests potential involvements of the vestibular structures in the brainstem in this disorder. VEMP may assist in identifying the SCA patients with a risk of unpredicted falls and preventing fall-related injuries. Since the correlation does not necessarily imply causation, additional large-scale studies are required for further elucidation of this association.

## Data Availability

Anonymized data will be shared by request from any qualified investigator.
